# *Notes from the Field:* Enterovirus A71 Neurologic Disease in Children — Colorado, 2018

**DOI:** 10.15585/mmwr.mm6736a5

**Published:** 2018-09-14

**Authors:** Kevin Messacar, Alexis Burakoff, W. Allan Nix, Shannon Rogers, M. Steven Oberste, Susan I. Gerber, Emily Spence-Davizon, Rachel Herlihy, Samuel R. Dominguez

**Affiliations:** ^1^Department of Pediatrics, University of Colorado School of Medicine, Aurora, Colorado; ^2^Department of Neurology, University of Colorado School of Medicine, Aurora, Colorado; ^3^Children’s Hospital Colorado, Aurora, Colorado; ^4^Epidemic Intelligence Service, CDC; ^5^Colorado Department of Public Health and the Environment, ^6^Division of Viral Diseases, CDC.

On May 10, 2018, the Colorado Department of Public Health and Environment (CDPHE) was notified by Children’s Hospital Colorado (CHCO) of an increase in pediatric cases of meningitis and encephalitis in which patients tested positive for enterovirus (EV). CDPHE surveillance data for May 2018 showed a 2.75-fold increase in encephalitis of unknown etiology compared with the 5-year (May 2013–2017) average; this coincided with a threefold rise in enterovirus/rhinovirus (EV/RV) detections from clinical testing at CHCO during the same period. Specimens from children with neurologic disease were tested by EV reverse transcription–polymerase chain reaction (RT-PCR) at CHCO and VP1 sequencing at CDC (*1*). As of August 26, 2018, EV-A71 was identified in 34 children with neurologic disease. This report describes the clinical, laboratory, and radiologic findings for the first 13 children identified with EV-A71 neurologic disease for whom complete information is available.

Patients with EV-A71 central nervous system (CNS) infection had symptom onset during March 10–June 5, 2018; median age was 13 months (range = 10 days–35 months); 11 were male. Twelve had meningitis, nine had encephalitis, and three had acute flaccid myelitis (AFM). All 13 children had fever and irritability; three developed lesions typical of hand, foot, and mouth disease. Neurologic signs included encephalopathy (seven), ataxia (seven), myoclonus (six), limb weakness (four), cranial nerve deficits (two), and seizures (one). Nine of 10 children with a cerebrospinal fluid (CSF) specimen analyzed had a pleocytosis (median white blood cell count = 106 cells/*μ*L, range = 17–698 [normal = 0–5]). Six of eight children who had brain imaging results had abnormalities; five were in the brainstem, three in the cerebellum, and three in the spinal cord. All 13 children had EV-A71 identified in nasopharyngeal, pharyngeal, or rectal specimens. However, only two of 11 children whose CSF was tested had a specimen positive for enterovirus by pan-EV RT-PCR; one of two was available for typing and was identified as EV-A71. All 13 children were hospitalized (median = 5 days; range = 1–23 days), and four required intensive care. The three children who received an AFM diagnosis had residual limb weakness at discharge. All children survived.

EV-A71 can cause hand, foot, and mouth disease and neurologic disease, primarily among children aged <5 years (*2*,*3*). Common manifestations include a febrile illness with lesions on the palms, soles, oral mucous membranes, or perineum; and aseptic meningitis. Severe CNS EV-A71 infection can cause brainstem encephalitis leading to cardiopulmonary collapse and polio-like AFM (*4*). EV-A71 epidemics have occurred in the Asian-Pacific region since the late 1990s (*5*). Since the 1980s, the National Enterovirus Surveillance System has detected seasonal endemic EV-A71 activity in the United States; EV-A71 accounts for <1% of typed EVs (*3*). Limited, regional U.S. outbreaks have occurred sporadically in an unpredictable pattern; factors causing year-to-year circulation have not been identified (*3*,*6*). Peak U.S. circulation of EVs, including EV-A71, usually occurs during June–October (*3*,*6*). Although associated with neurologic disease, EV-A71 is uncommonly detected in CSF and is more frequently identified in respiratory and fecal specimens (*7*). In similar EV-A71 outbreaks in Colorado during 2003 and 2005, EV-A71 CNS infection was identified in 16 children (eight in each cluster); 11 children recovered fully, four had residual limb paralysis, and one child died (*7*). At this time, no other clusters of EV-A71 neurologic disease have been reported to CDC in 2018.

This investigation highlights the importance of testing nonsterile sites when CNS disease associated with EV is suspected and CSF is negative. Furthermore, health care providers should consider EV-A71 as an etiology when febrile patients display myoclonus, ataxia, or limb weakness. CDPHE has alerted Colorado health care providers to the EV-A71 outbreak and requested reports of EV meningitis and encephalitis, in addition to routine AFM surveillance.

## References

[1] Nix WA, Oberste MS, Pallansch MA. Sensitive, seminested PCR amplification of VP1 sequences for direct identification of all enterovirus serotypes from original clinical specimens. J Clin Microbiol 2006;44:2698–704. 10.1128/JCM.00542-06 16891480 PMC1594621

[2] Ooi MH, Wong SC, Lewthwaite P, Cardosa MJ, Solomon T. Clinical features, diagnosis, and management of enterovirus 71. Lancet Neurol 2010;9:1097–105. 10.1016/S1474-4422(10)70209-X 20965438

[3] Khetsuriani N, Lamonte-Fowlkes A, Oberst S, Pallansch MA. Enterovirus surveillance—United States, 1970–2005. MMWR Surveill Summ 2006;55(No. SS-8). 16971890

[4] Huang CC, Liu CC, Chang YC, Chen CY, Wang ST, Yeh TF. Neurologic complications in children with enterovirus 71 infection. N Engl J Med 1999;341:936–42. 10.1056/NEJM199909233411302 10498488

[5] Solomon T, Lewthwaite P, Perera D, Cardosa MJ, McMinn P, Ooi MH. Virology, epidemiology, pathogenesis, and control of enterovirus 71. Lancet Infect Dis 2010;10:778–90. 10.1016/S1473-3099(10)70194-8 20961813

[6] Pons-Salort M, Oberste MS, Pallansch MA, The seasonality of nonpolio enteroviruses in the United States: patterns and drivers. Proc Natl Acad Sci U S A 2018;115:3078–83. 10.1073/pnas.1721159115 29507246 PMC5866597

[7] Pérez-Vélez CM, Anderson MS, Robinson CC, Outbreak of neurologic enterovirus type 71 disease: a diagnostic challenge. Clin Infect Dis 2007;45:950–7. 10.1086/521895 17879907

